# SinEx DB 2.0 update 2020: database for eukaryotic single-exon coding sequences

**DOI:** 10.1093/database/baab002

**Published:** 2021-01-28

**Authors:** R Jorquera, C González, P T L C Clausen, B Petersen, D S Holmes

**Affiliations:** Center for Bioinformatics and Genome Biology, Fundacion Ciencia & Vida, Zañartu 1482, Ñuñoa Santiago 7780132, Chile; Laboratorio Medicina Traslacional, Fundación Arturo López Pérez, José Manuel Infante 805, Providencia, Santiago 7500691, Chile; Center for Bioinformatics and Genome Biology, Fundacion Ciencia & Vida, Zañartu 1482, Ñuñoa Santiago 7780132, Chile; Centro de Genómica y Bioinformática, Universidad Mayor, Camino la pirámide 5750, Huechuraba, Santiago 8580745, Chile; Department of Global Surveillance, Technical University of Denmark, Kemitorvet building 204, 2800 Kgs. Lyngby, Denmark; Section for Evolutionary Genomics, The GLOBE Institute, University of Copenhagen, Hovedstaden, Øster Voldgade 5–7, Copenhagen 1350, Denmark; Centre of Excellence for Omics-Driven Computational Biodiscovery (COMBio), AIMST University, Batu 3 1/2, Jalan Bukit Air Nasi, 08100 Bedong, Kedah, Malaysia; Center for Bioinformatics and Genome Biology, Fundacion Ciencia & Vida, Zañartu 1482, Ñuñoa Santiago 7780132, Chile; Centro de Genómica y Bioinformática, Universidad Mayor, Camino la pirámide 5750, Huechuraba, Santiago 8580745, Chile

## Abstract

Single-exon coding sequences (CDSs), also known as ‘single-exon genes’ (SEGs), are defined as nuclear, protein-coding genes that lack introns in their CDSs. They have been studied not only to determine their origin and evolution but also because their expression has been linked to several types of human cancers and neurological/developmental disorders, and many exhibit tissue-specific transcription. We developed SinEx DB that houses DNA and protein sequence information of SEGs from 10 mammalian genomes including human. SinEx DB includes their functional predictions (KOG (euKaryotic Orthologous Groups)) and the relative distribution of these functions within species. Here, we report SinEx 2.0, a major update of SinEx DB that includes information of the occurrence, distribution and functional prediction of SEGs from 60 completely sequenced eukaryotic genomes, representing animals, fungi, protists and plants. The information is stored in a relational database built with MySQL Server 5.7, and the complete dataset of SEG sequences and their GO (Gene Ontology) functional assignations are available for downloading. SinEx DB 2.0 was built with a novel pipeline that helps disambiguate single-exon isoforms from SEGs. SinEx DB 2.0 is the largest available database for SEGs and provides a rich source of information for advancing our understanding of the evolution, function of SEGs and their associations with disorders including cancers and neurological and developmental diseases.

**Database URL:** http://v2.sinex.cl/

## Introduction

Eukaryotic genes are usually interrupted by intragenic, non-protein-coding regions termed ‘introns’ that are removed by RNA splicing during maturation of the final RNA product. However, >2000 protein-coding genes in the human genome have been shown to lack introns and have been termed ‘single-exon genes’ (SEGs), defined as nuclear, protein-coding genes that lack introns in their coding sequences (CDSs) (1, 2). This definition excludes genes that generate functional RNAs such as tRNA, rRNA and long non-coding RNAs (2).

There is evidence in literature that expression of many human SEGs is linked to several types of cancers (3–5) and neurological and developmental disorders (6–8). In addition, the expression of some SEGs has been shown to be tissue specific (8, 9). These discoveries highlight the importance of studying SEGs to uncover properties and evolutionary trajectories that underlie their relationships with pathologies and normal phenotypes. In order to facilitate the discovery of novel SEGs and to reveal new functional relationships, we created SinEx DB (1).

The updated SinEx DB 2.0 has increased the number of genomes interrogated from 10 to 60 and has expanded the phylogenetic representation from only mammals to incorporate other eukaryotes including fungi, protists and terrestrial plants. Additional improvements in SinEx DB 2.0 include new functional assignations of SEGs using InterPro database 69.0 (10) and InterPro Scan 5.3 (11) including GO functional categorizations.

SinEx DB 2.0 also addressed an important emerging problem. Many SEGs are being confused with single-exon isoforms (SEIs). SEIs arise from alternative splicing of multi-exonic genes in which only one exon is processed (1). SinEx DB 2.0 was implemented using an improved SEG identification pipeline that allows the identification and storage of SEGs separately from SEIs.

SinEx DB 2.0 is the largest database for SEGs built to date. It is anticipated that it provides a rich, curated source of information for advancing our understanding of the evolution and function of SEGs and their association with disorders including cancers and neurological and developmental diseases. It can also be used as a comparative platform for annotating SEGs in eukaryotic genomes.

## Database construction

**Figure 1.**
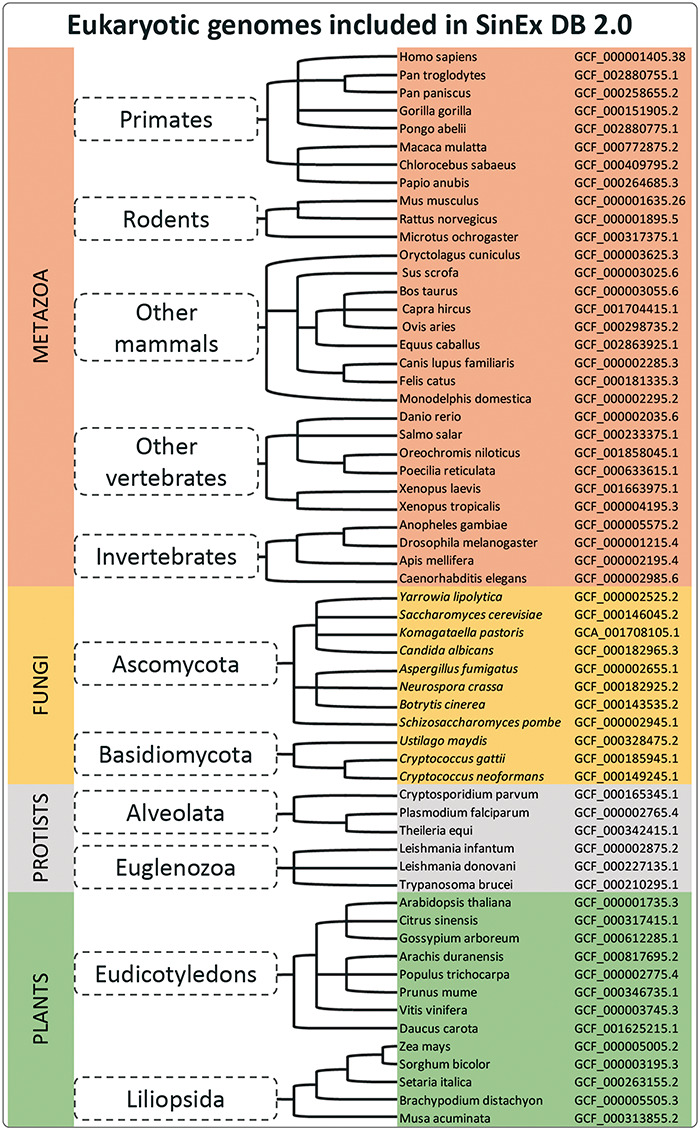
A simplified cladogram of genome assemblies downloaded from NCBI database. The updated SinEx DB 2.0 has increased the number of genomes interrogated from 10 to 60 and has expanded the phylogenetic representation from only mammals to incorporate other eukaryotes including fungi, protists and terrestrial plants.

Sixty sequenced and annotated eukaryotic genomes, assembled at a chromosome level, were downloaded from GenBank (12) at the FTP site on the NCBI web page (ftp://ftp.ncbi.nlm.nih.gov/genomes/). A complete list of the genome assemblies downloaded for the database construction is shown in Figure 1.

CDS gene identifiers in the GenBank-format chromosome files were selected and classified into SEGs and multi-exon genes (MEGs) as described previously (1). A minimum Open Reading Frame (ORF)/CDS size of 30 nucleotides was used for the selection of sequences. CDSs containing the ‘/pseudo’ tag (annotated as inactive pseudogenes) were binned separately. CDSs located on the same strand and overlapping with at least one exon of a MEG were identified, classified and binned as ‘SEIs’, consistent with new ontology definitions for single-exon sequences (2). Redundancy filters are implemented to minimize the entry of duplicate sequences (e.g. same gene ID and same coordinates). Unique entries are provided with a unique tag in the FASTA header to facilitate further investigations into functional associations and phylogenetic tree construction. Functional classifications of SEGs were made using InterPro database 69.0 (10) and InterPro Scan software 5.3 (11).

Information is stored in a relational database built with MySQL Server 5.7. The system back-end was built in NodeJS 10.0 with Express as a framework and the front-end built in VueJS and Bootstrap 4.0. Data transfer in SinEx DB 2.0 is made through API REST (JSON) using NodeJS instead of PHP as used in SinEx DB 1.0. This improves the performance, allowing multiple parallel processing (many users at a time). Having the system split into two layers (back-end and front-end) allows the data from the MySQL database to be transferred to and rendered in the user’s browser in a lighter format (JSON) via REST services. Thus, all possible parallel jobs can be run in an efficient and rapid way.

The complete dataset of SEGs and their functional assignations in FASTA and gff3 files, respectively, are available for downloading.

## Results/data content

SinEx DB 2.0 provides information regarding the occurrence, properties and genomic distribution of approximately 213 000 SEGs (compared to 31 624 SEGs in SinEx DB 1.0) out of a total of about 1 848 000 annotated CDSs (248 152 total CDSs in SinEx DB 1.0) from 60 completely sequenced eukaryotic genomes. CDSs identified as SEIs were binned separately and their chromosome location, sequence accession number, gene and exon associated with their transcription data are available for downloading in tsv file format.

SinEx DB 2.0 contains SEGs from 20 mammalian genomes (8 primates including *Homo sapiens*, 3 rodents and 9 other mammals), 6 other vertebrates such as *Danio rerio* and *Xenopus tropicalis* and 4 invertebrates including *Drosophila melanogaster* (Figure 1), for a total of 30 species from the division Metazoa. SinEX DB 2.0 also contains 30 genomes from three other divisions, namely: 11 Fungi (including Ascomycetes and Basidiomycetes); 6 Protists (including Alveolata and Euglenozoa) and 13 Plants (including Eudicotyledons and Liliopsida) (Figure 1).

## Web interface

There are two ways to access SinEx DB 2.0 data via the web interface: (i) by interrogating a protein sequence as a query in BLASTP (13) against the in-house SinEx DB and (ii) by performing an advanced search using ‘genome’, ‘chromosome number’, ‘protein name’, ‘gene symbol’, ‘GO ID’ or ‘GO name’. The search by protein name is not case-sensitive but is sensitive to different spelling. Hot-links to NCBI sequence accession entries (12) and to gene ontology annotation data (14, 15) were included for all sequences within the SinEx DB 2.0 web interface. Protein sequences of SEGs in FASTA format as well as SEG functional assignation and SEI information from 60 eukaryotic genomes included in SinEx DB 2.0 are downloadable. A section of statistical information of occurrence of SEGs in eukaryotic genomes and a frequently asked questions (FAQs) section to facilitate user’s recovery of data are also available in the web page.

## Conclusion

SinEx DB 2.0 provides an opportunity to address questions regarding the occurrence, distribution, evolution and function of SEGs in 60 diverse high-quality eukaryotic genomes representing animals, plants, fungi and protists. SinEx DB 2.0 complements existing databases such as Retrogene DB (16), Pseudogene DB (17) and APPRIS (18). It could also be used as a comparative platform for annotating single-exon CDSs in mammalian genomes.

## Future perspectives

It is proposed to update SinEx DB once a year with annotated SEGs from additional completely sequenced eukaryotic genomes, ranging from unicellular eukaryotes to mammals. Future versions of the database will incorporate transcriptomic data from different genomes, in order to distinguish between SEGs with UTR (UnTranslated Region) introns (uiSEGs) from those SEGs without (intronless genes).

We propose that SEGs from different and diverse genomes available in future versions of SinEx DB could be integrated with relevant platforms with single-exon architecture such as Retrogene DB (16), Pseudogene DB (17) and APPRIS (18) for SEIs.

## Availability and requirements

SinEx DB 2.0 is freely and publicly available at http://v2.sinex.cl/ and the complete dataset is available for download.
